# Genetic and histopathological analysis of transverse testicular ectopia without persistent Müllerian duct syndrome: two case reports

**DOI:** 10.1186/s13256-020-02559-7

**Published:** 2020-12-01

**Authors:** Takashi Nagai, Kentaro Mizuno, Masayuki Usami, Hidenori Nishio, Taiki Kato, Akihiro Nakane, Daisuke Matsumoto, Satoshi Kurokawa, Hideyuki Kamisawa, Tetsuji Maruyama, Takahiro Yasui, Yutaro Hayashi

**Affiliations:** 1 Department of Nephro-urology, Nagoya City University Graduate School of Medical Sciences, Nagoya, Japan; 2 Department of Pediatric Urology, Nagoya City University Graduate School of Medical Sciences, 1 Kawasumi, Mizuho-cho, Mizuho-ku, Nagoya, 467-8601 Japan; 3 Department of Urology, Toyota Kosei Hospital, Toyota, Japan; 4 Education and Research Center for Community Medicine, Nagoya City University Graduate School of Medical Sciences, Nagoya, Japan; 5 Education and Research Center for Advanced Medicine, Nagoya City University Graduate School of Medical Sciences, Nagoya, Japan

**Keywords:** Transverse testicular ectopia, Anti-Müllerian hormone, Persistent Müllerian duct syndrome

## Abstract

**Background:**

Transverse testicular ectopia (TTE) is a rare anomaly in which both testes descend through a single inguinal canal into the same hemiscrotum. Although almost 20–50% of patients with TTE exhibit persistent Müllerian duct syndrome (PMDS) and many genetic analyses have been performed, no reports have described the genes contributing to TTE without PMDS. Here, we report two cases of TTE without PMDS using immunohistochemical staining and genetic analysis.

**Case presentation:**

Two Asian patients with TTE without PMDS were subjected to orchiopexy. We performed testicular biopsies during operation and obtained blood samples before the operation. Testicular tissues were stained for c-kit, placental alkaline phosphatase (PLAP), and undifferentiated embryonic cell transcription factor 1 (UTF1) to evaluate the presence of intratubular malignant germ cells. Additionally, we performed polymerase chain reaction-based direct sequencing to identify single nucleotide polymorphisms in genes associated with regression of the Müllerian duct and testicular descent (that is, anti-Müllerian hormone [*AMH*], AMH receptor 2 [*AMHR2*], insulin-like 3 [*INSL3*], and relaxin family peptide receptor 2 [*RXFP2*]). The three-dimensional structures of proteins were predicted using SWISS-MODEL. In immunohistochemical analysis, c-kit and UTF1 were positive, whereas PLAP was negative in three testicular tissue samples from the two patients. These features were also detected on the unaffected side. In variant analysis, common missense variants in the *AMH* gene (g.365G>T; c.165G>T; p.Ser49Ile [rs10407022]) were observed. All variants in *INSL3* and *RXFP2* genes were intronic or silent.

**Conclusions:**

Because UTF1, a specific marker of spermatogonial stem cell activity, was expressed in both the affected and unaffected sides in the testicular tissues of two patients, the risk of malignancy may be high in these patients. Although the etiology of TTE without PMDS remains unclear, our variant analysis results were consistent with previous reports, and variants in the *AMH* gene (rs10407022) may contribute to the specific phenotype of TTE without PMDS.

## Background

Ectopic testis is a congenital anomaly in which testes are located outside of the normal path of descent, that is, in the superficial inguinal pouch, supurapubic region, femoral region, base of penis, or opposite side of hemiscrotum [1]. Transverse testicular ectopia (TTE) is a rare anomaly in which both testes descend through a single inguinal canal into the same hemiscrotum. In the absence of testicular tumors, the treatment for TTE is orchiopexy. However, patients with TTE have a higher risk of testicular tumors and cryptorchidism; indeed, the incidence of testicular tumors in patients with TTE is 18% [2], which is higher than that observed in patients with isolated cryptorchidism.

The pathogenesis of TTE involves persistent Müllerian ducts, fusion of the vas deferens, and primary gubernacular defects. Approximately 20–50% of patients with TTE also exhibit persistent Müllerian duct syndrome (PMDS), which is strongly associated with TTE [3–5]. The etiology of PMDS is explained by inadequate Müllerian suppression of Müllerian ducts owing to the glycoprotein anti-Müllerian hormone (AMH). Specific variants in the genes encoding AMH and its receptor AMHR2 have been reported in patients with PMDS [6]. Although AMH and AMHR2 have been reported to be responsible for PMDS, no reports have described the genes contributing to TTE without PMDS. However, some authors have described relationships between anomalies in the gubernaculum, that is, congenital fascial bands [7] and anomalous distal insertion [8].

Various processes are involved in descent of the testes, and insulin-like 3 (INSL3) protein is known to play a role in descent of the testes in the transabdominal phase and growth of the gubernaculum [9]. INSL3 is a member of the insulin-like hormone family and is exclusively synthesized in the gonads. INSL3 and its receptor relaxin family peptide receptor 2 (RXFP2) play important roles in descent of the testes during the transabdominal phase, and single-nucleotide polymorphisms in the *INSL3* and *RXFP2* genes are risk factors for cryptorchidism [10].

Despite these previous studies, few reports have described the histology or genetics of TTE, likely because of the very low incidence of this condition. Here, we report two cases of TTE without PMDS. We investigated their testicular histology and genetic alterations in the *AMH*, *AMHR2*, *INSL3*, and *RXFP2* genes to elucidate the pathological features and genetic etiology of the testes in these patients using immunohistochemistry and direct sequencing analyses.

## Case presentation

### Case 1

**Fig. 1:**
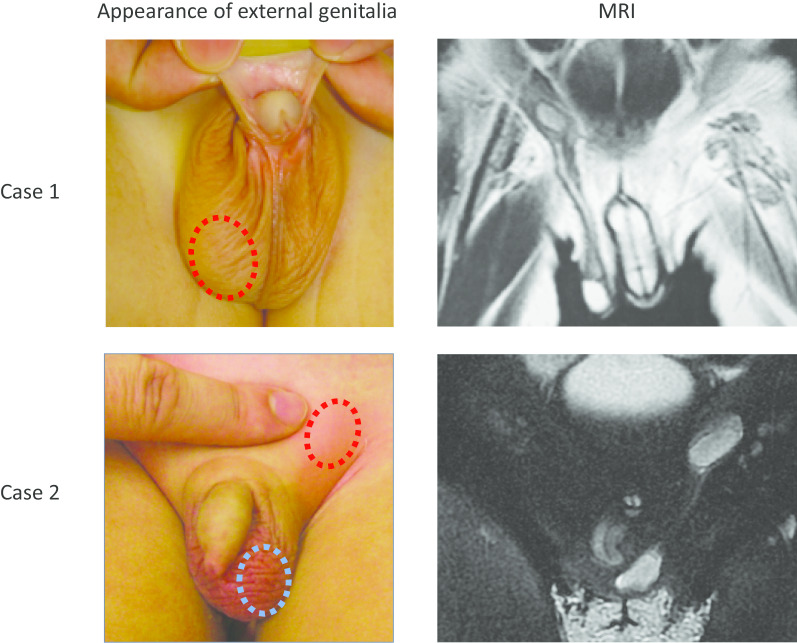
Appearance of external genitalia and MRI findings in cases 1 and 2. Dotted circles indicate palpable testes

This Asian patient was naturally delivered at 39 weeks, 0 days via normal labor and had no siblings. The birth weight was 2426 g, and Apgar scores were 9(1′) and 9(5′). At birth, the baby was diagnosed with bilateral nonpalpable testes and hypospadias. Chromosomal and genetic tests showed that the karyotype was 46,XY and that the *SRY* gene was positive. Thus, the baby was brought up as a boy. At 4 months old, his right testis was palpable in his right scrotum. Because his left testis was still nonpalpable, he was referred to our hospital. Ultrasonography and magnetic resonance imaging (MRI) revealed that left testis was located around his right groin (Fig. 1). Thus, we diagnosed TTE and performed laparoscopic left orchiopexy at 15 months of age. Preoperative serum hormone levels are shown in Table 1.

**Table 1:** Patients’ characteristics at surgery

Case no.	Age (months)	Laterality	Other anomalies	Karyotype	LH (mIU/mL)	FSH (mIU/mL)	Testosterone (ng/mL)	AMH (pmol/L	Treatment
1	15	Left	Hypospadias	46,XY	< 0.1	2.5	< 0.03	850	Laparoscopic orchiopexy
2	25	Right	None	46,XY	0.6	0.1	< 0.03	1842	Transseptal orchiopexy

According to our previous report [11], conventional laparoscopy uncovers the left abdominal testis just above the right internal ring. The right processus vaginalis was opened. We could not confirm the existence of any Müllerian duct derivatives. After sufficient mobilization, the left testis was delivered into the subdartos pouch of the left scrotum. The left testis was fixed in the scrotum using a 5–0 polyglactin suture, and at that time, testicular biopsy was also performed with permission from the patient’s parents. Subsequently, at 22 months old, we performed repair of hypospadias. There were no postoperative complications, and both testes were located in the lower scrotum. Both testes were palpable in their respective scrotums without testicular ascent or atrophy at 8 years postoperatively.

## Case 2

This patient was born at 39 weeks, 3 days via spontaneous delivery. The Asian patient had no siblings, and the birth weight was 3306 g. At 1 month of age, he was found to lack testis in the right scrotum during a medical checkup. He was followed up by a practicing physician, but then referred to our hospital because of spontaneous descent of the right testis at 18 months of age; his left testis was located in his right scrotum, and a small mass was palpable in his left groin. MRI revealed that the right testis was located around his left lower abdomen (Fig. 1), and he was then diagnosed with TTE. His karyotype was 46,XY, and he was positive for the *SRY* gene. Endocrinological examination revealed that all parameters were within normal limits as appropriate for his age.

First, we performed conventional laparoscopy, and the right abdominal testis was detected just above the left internal ring with left patent processus vaginalis. The bilateral vas deferens and spermatic vessels ran toward the left internal ring and left testis without Müllerian duct derivatives. Although we attempted to fix his right testes via the right groin into the right scrotum, right orchiopexy with a transseptal approach [12] was required to avoid injury to the vas deferens and vessels. Both testes were palpable in their respective scrotums without testicular ascent or atrophy at 5 years postoperatively. The characteristics of the two patients at surgery are shown in Table 1.

To evaluate the histopathology of their testes and genetic etiology in these patients with TTE, we performed immunohistological examination and gene variant analysis. We obtained informed consent from the two patients’ families for participation in sample collection and analysis. We performed testicular biopsy during surgery and obtained three testicular tissues (left testis from case 1; bilateral testes from case 2). The tissues were fixed in 4% paraformaldehyde for histological examination. Blood samples were also collected prior to operation for gene analysis. Studies using human testicular tissue and genomic DNA were approved by the institutional review board of Nagoya City University Hospital (approved number: 84).

Testicular tissues fixed in 4% paraformaldehyde were embedded in paraffin and sectioned at 5 μm thickness. Histopathological examination of hematoxylin-eosin staining and immunohistochemical analysis were performed using serial sections. Signals were detected using anti-c-Kit antibodies (1:500; catalog no. A4502; Dako, Tokyo, Japan), anti-placental alkaline phosphatase (PLAP) antibodies (1:50; catalog no. M7191; Dako), and anti-undifferentiated embryonic cell transcription factor 1 (UTF1) antibodies (1:1000; catalog no. MAB4337; Merck Millipore, Darmstadt, Germany), as described previously [13]. Briefly, after blocking nonspecific binding with 5% skim milk in phosphate-buffered saline, 5-μm-thick sections were incubated overnight at 4 °C. Signals were detected with an avidin-biotinylated enzyme complex system, using a VECTASTAIN ABC Kit (Vector Laboratories, Burlingame, CA, USA) and anti-rabbit IgG (catalog no. PK-6101; Vector Laboratories), according to the manufacturer’s instructions.

Genomic DNA was extracted from leukocytes isolated from patient’s peripheral blood using a Wizard genomic DNA purification kit (Promega, Madison, WI, USA), according to the manufacturer’s instructions, as previously reported [14]. The purity of the extracted DNA was determined by measurement of absorbance and evaluation of gel electrophoresis results. The primers for amplification were designed based on the specific deoxyribonucleic acid (DNA) sequences (Additional file 1). Amplification of exons was performed by PCR using sequence-specific primers. PCR products were sequenced using an ABI BigDye Terminator ver3.1 Cycle Sequencing Kit (Thermo Fisher Scientific, Waltham City, MA, USA) with an ABI 3730 DNA Analyzer (Applied Biosystems, Foster City, CA, USA) based on the primer sets used for sequencing (Additional file 2). Variants were identified by comparisons with RefSeq in the NCBI database (https://www.ncbi.nlm.nih.gov/). The three-dimensional structures of proteins were predicted using SWISS-MODEL (https://swissmodel.expasy.org/).

**Fig. 2:**
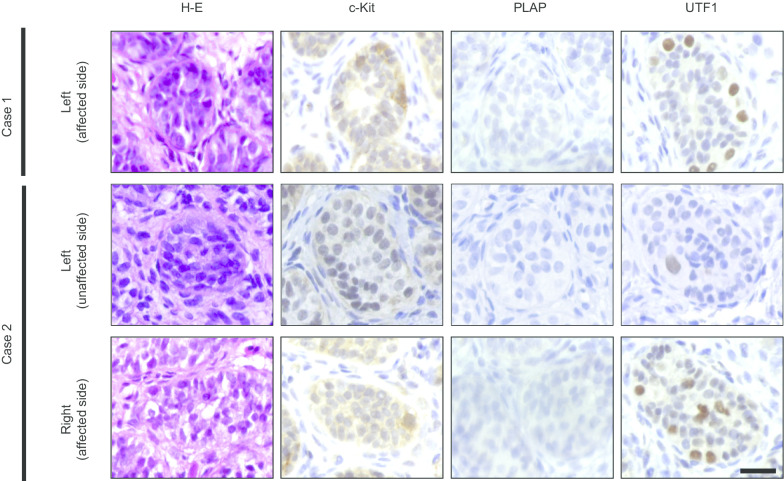
Immunohistochemical staining of testicular tissues from the two cases. In case 2, testicular biopsy of the unaffected side was performed after obtaining approval from the patient’s parents. Notably, c-kit and UTF1 were positive in spermatogonia and spermatocytes in all samples, whereas PLAP was negative. H-E: hematoxylin-eosin, c-kid, PLAP: placental alkaline phosphatase, UTF1: undifferentiated embryonic cell transcription factor 1. Scale bar: 30 μm

**Fig. 3:**
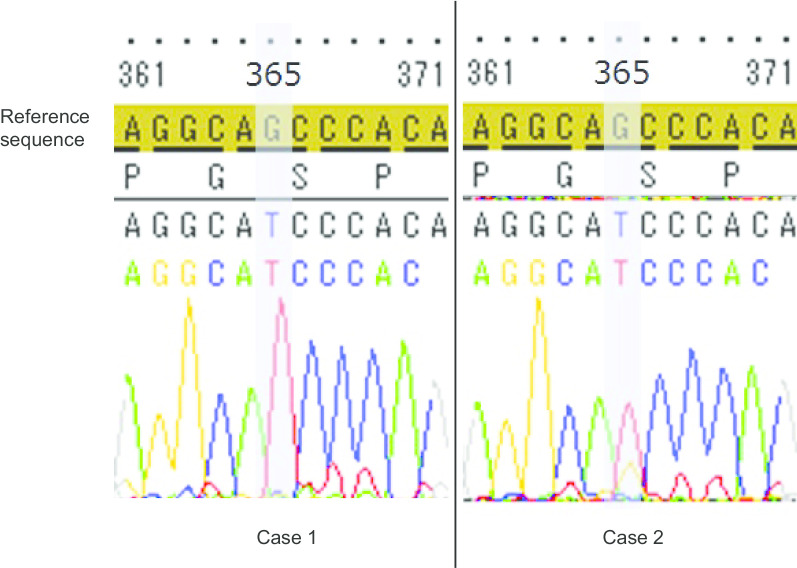
Partial chromatograms the *AMH* gene variant in cases 1 and 2. The base change g.365G > T led to the missense variant in exon 1

**Fig. 4:**
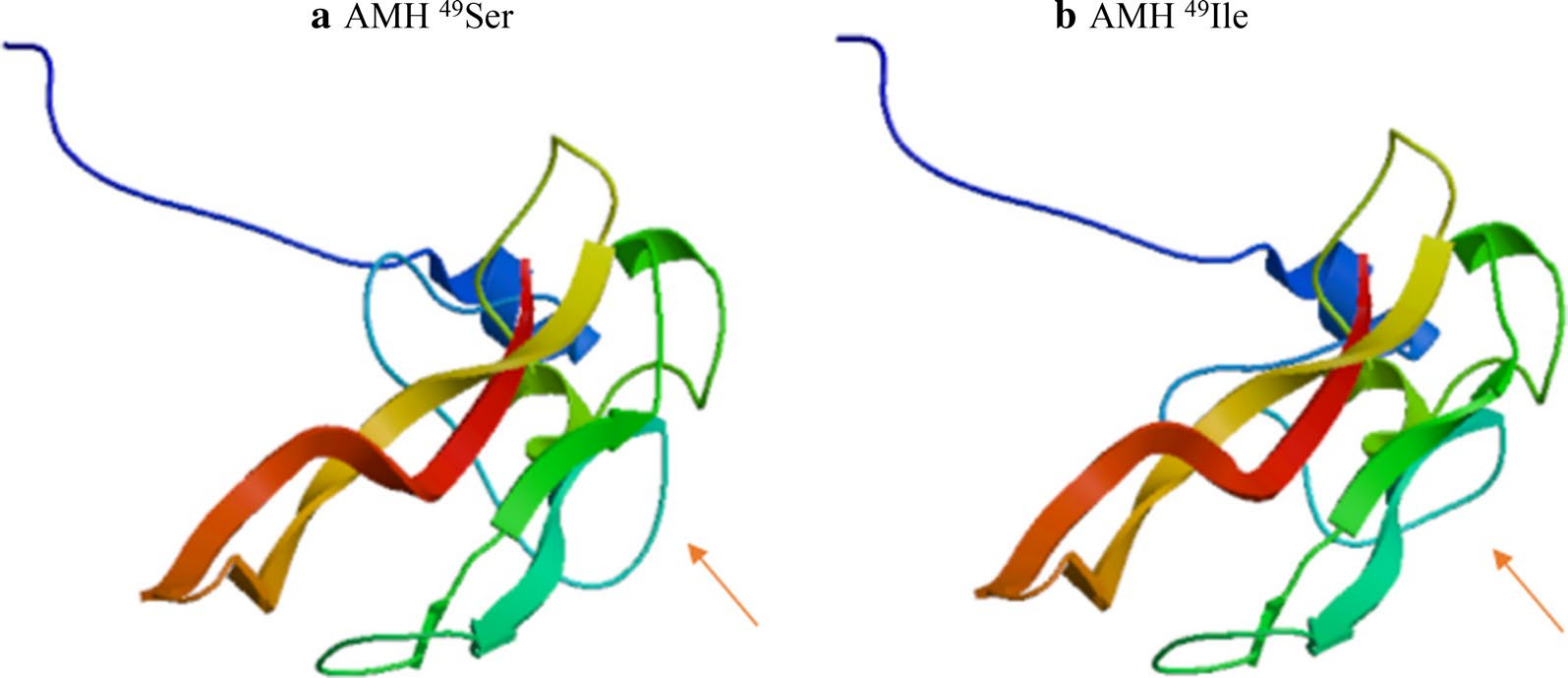
Three-dimensional prediction of AMH protein by SWISS-MODEL. Protein models of AMH ^49^Ser **a** and AMH ^49^Ile **b** are shown. The change in loop structure is shown (arrow)

Immunohistochemical staining of testicular tissue is shown in Fig. 2. In three samples from the two patients, c-kit was positive, whereas PLAP was negative. Thus, a definitive diagnosis of intratubular malignant germ cells could not be made. UTF1, a marker of undifferentiated spermatogonial stem cells, was positive in these samples, even in the testes of the unaffected side in case 2. The results of PCR-based direct sequencing of *AMH*, *AMHR2*, *INSL3*, and *RXFP2* are shown in Table 2. We observed common variants in two cases. The common variants in the *AMH* gene were g.365G>T and g.1416G>A; g.365G>T was a missense variant in exon 1 (c.165G>T), changing Ser to Ile (p.Ser49Ile; Fig. 3), and g.1416G>A was present in intron 2. With respect to g.365G>T (c.165G>T; p.Ser49Ile) in the *AMH* gene, protein structure prediction by Swiss-Model indicated that the loop structure was altered (Fig. 4). There were no common variants in the *AMHR2* gene. A common variant, g.4983G>A, was observed in the *INSL3* gene; however, this variant was within an untranslated region. In the *RXFP2* gene, eight common variants were observed in the intronic region and in the 3′ untranslated region, and one variant was observed in exon 12 (g.46869A>G; c.963A>G). The variant in exon 12 was a nonsense variant.

**Table 2:** Genetic variants in *AMH*, *AMHR2*, *INSL3*, and *RXFP2*

Gene symbol	Case 1	Case 2	Mutation type
*AMH*	g.365G>T		Missense (Ser49Ile)
	g.1357G>A		–
	g.1416G>A		–
*AMHR2*	–	g.5096C>T	–
*INSL3*	g.4983G>A	g.4983G>A	Intronic
	g.4727C>T		–
*RXFP2*	g.21848A>T	g.21848A>T	Intronic
	g.25883T>C	g.25883T>C	Intronic
	g.26523_26524insA	g.26523_26524insA	Intronic
	g.35339A>G	g.35339A>G	Intronic
	g.42015_42016insC	g.42015_42016insC	Intronic
	g.42610C>T	g.42610C>T	Intronic
	g.46869A>G	g.46869A>G	Silent (c.963A>G)
	g.57973T>C	g.57973T>C	Intronic
	g.63040A>G	g.63040A>G	Intronic

## Discussion and conclusions

We reported two cases of TTE without PMDS. Furthermore, we suggested the tumor risk of the patients with TTE and necessity of long-term follow-up for both testes based on genetic and histopathological analysis. To our knowledge, this is the first report regarding genetic and histopathological features focused on TTE without PMDS.

TTE is a rare form of testicular ectopia in both testes existing in the same side of the hemiscrotum. Since the first case was reported by Von Lenhossek in 1886, more than 260 cases have been reported in the English literature [12]. According to previous reports [15, 16], the standard treatment is orchiopexy; when accompanied by testicular tumors, orchiectomy is performed. TTE is accompanied by various abnormalities, such as PMDS, inguinal hernia, and hypospadias. Here we reported two cases of TTE without PMDS and identified common pathological features in their testes, including positivity for c-kit and the presence of UTF1-positive germ cells. Notably, these features were also detected on the unaffected side. Additionally, we identified a common missense variant in the exonic region of the *AMH* gene (g.365G>T; c.165G>T [rs10407022]), providing important insights into the genetic basis of TTE.

The incidence of testicular tumors in patients with TTE is 18% [2], which is equivalent to that of patients with abdominal testes and higher than that in healthy individuals and patients with inguinal testes. In this study, we evaluated whether our cases exhibited intratubular malignant germ cells by immunohistochemical staining of testicular tissue. Notably, both cases were positive for c-kit, negative for PLAP, and positive for UTF1, a specific marker of spermatogonial stem cell activity [17] that can be used to diagnose testicular tumors [18]. Thus, we identified undifferentiated spermatogonial stem cells in the testis of patients with TTE. Interestingly, both affected and unaffected testes were positive for UTF1 in case 2. Thus, we assumed that testicular tumors may appear in the contralateral testis in the future. To the best of our knowledge, this is the first report to consider the possibility of malignancy in the testes of patients with TTE by immunohistochemical staining.

Concerning the etiology of TTE, several hypotheses have been proposed, as follows, including defective implantation of the gubernaculum, obstruction of the inguinal ring, adherence and fusion of the developing Wolffian ducts, and existence of derivatives from the Müllerian duct (PMDS) [15]. Although many reports have described genetic variations in TTE with PMDS [19, 20], few studies have reported TTE without PMDS. Testicular descent is a stepwise process that involves various genes. Zimmermann et al. [21] reported that complete loss of gubernacular attachment, transverse ectopia, and/or torsion were observed in transgenic mice null for *Insl3* and that INSL3 regulated gubernaculum development through its receptor, RXFP2, during the transabdominal descent of the testes [9]. In the current study, we investigated gene variants associated with testicular descent (*INSL3* and *RXFP2*), but only one silent variant (*RXFP2* c.963A>G) was observed among two patients, and the other variants were within the intronic region. Because *INSL3* (1.8%) and *RXFP2* (2.9%) gene variants are rarely observed in idiopathic cryptorchidism [22] and there are no reports of these gene variants in TTE, it is likely that these gene variants are not associated with development of TTE.

On the basis of the presence of various associated anomalies, TTE can be classified into three types [23]: type 1, accompanied by hernia; type 2, accompanied by PMDS; type 3 associated with hypospadias, scrotal abnormalities, fused vas deferens, or testicular microlithiasis [15]. According to this classification, case 1 was type 1, and case 2 was type 3 TTE. Almost 20–50% of patients with TTE exhibit PMDS [2], and some reports have suggested that PMDS is involved in the pathogenesis of TTE. As reviewed by Picard et al. [20], several variants in the *AMH* and *AMHR2* genes have been reported in patients with TTE with PMDS [6, 19]. AMH is responsible for the regression of Müllerian ducts in male fetuses, and variants inactivating AMH or its receptor AMHR2 lead to PMDS. Although the cases in the current report were TTE without PMDS, a common variant in the *AMH* gene was observed, including the missense variant g.365G>T (c.165G>T; p.Ser49Ile). These findings suggested that abnormalities other than PMDS, for exampe, fused vas deferens, may be associated with variants in the *AMH* gene.

The identified variant (*AMH* c.165G>T; rs10407022) is known to be related to polycystic ovary syndrome (PCOS) [24]. Additionally, this variant has been shown to be associated with reduced incidence of PCOS and lower follicle numbers in women with PCOS. Kevenaar et al. also investigated the roles of the AMH signaling pathway in the pathophysiology of PCOS using a genetic approach [25]. They showed that the AMH Ile^49^Ser polymorphism, which was the same as the variant identified in the current study, contributes to the severity of the PCOS phenotypes. Moreover, they confirmed that the bioactivity of the AMH ^49^Ser protein was diminished compared with that of the AMH ^49^Ile protein using an in vitro study [25]. Although the relationship between TTE and PCOS is still unclear, these conditions are similar in that they are both characterized by abnormal gonadal morphology. More recently, Greiber et al. reported that the *AMH* genotype (rs10407022 T>G) is associated with higher serum AMH levels in prepubertal Danish boys [26]. The TT allele in our current two cases suggested that serum AMH levels may be lower compared with that in healthy boys. Although the spot concentration of serum AMH was normal in case 1, it was higher than that in age-matched healthy individuals in case 2 [27]. Thus, these findings may reflect negative feedback caused by lower AMH levels. As described above, we could not rule out that *AMH* gene variation could lead to a specific phenotype, TTE without PMDS, in the current cases. However, the number of cases was small, and further functional studies are needed to confirm these findings.

In conclusion, we reported two cases of TTE without PMDS and described the histological, immunological, and genetic features from these cases. Our findings suggested that patients with TTE may be at higher risk of testicular tumors on both the affected and unaffected sides. Thus, physicians should be careful to monitor this possibility in both testes. Although the etiology remains unclear, common missense variants in the *AMH* gene (g.365G>T; c.165G>T [rs10407022]) may affect the development of TTE without PMDS. Further studies with more patients are warranted.

## Supplementary information

**Additional file 1.** Primer set used for ploymerase chain reaction (PCR)

**Additional file 2.** Primer set used for sequencing

## Data Availability

The datasets generated during the current study are not publicly available because it is possible that individual privacy could be compromised.

## Abbreviations

AMH: Anti-Müllerian hormone
AMHR2: Anti-Müllerian hormone receptor type 2
INSL3: Insulin-like peptide 3
PCR: Polymerase chain reaction
PLAP: Placental alkaline phosphatase
PMDS: Persistent Müllerian duct syndrome
RXFP2: Relaxin/insulin-like family peptide receptor 2
TTE: Transverse testicular ectopia
UTF1: Undifferentiated embryonic cell transcription factor 1
