# Supplementary material for: Genetic and histopathological analysis of transverse testicular ectopia without persistent Müllerian duct syndrome: two case reports
Source: J Med Case Rep. 2020 Dec 1;14:233. doi: 10.1186/s13256-020-02559-7 (PMC7706043; doi:10.1186/s13256-020-02559-7)
Supplement: Supplementary file 1 — Additional file 1. Primer set used for ploymerase chain reaction (PCR) [file 13256_2020_2559_MOESM1_ESM.pptx]

## Slide 1
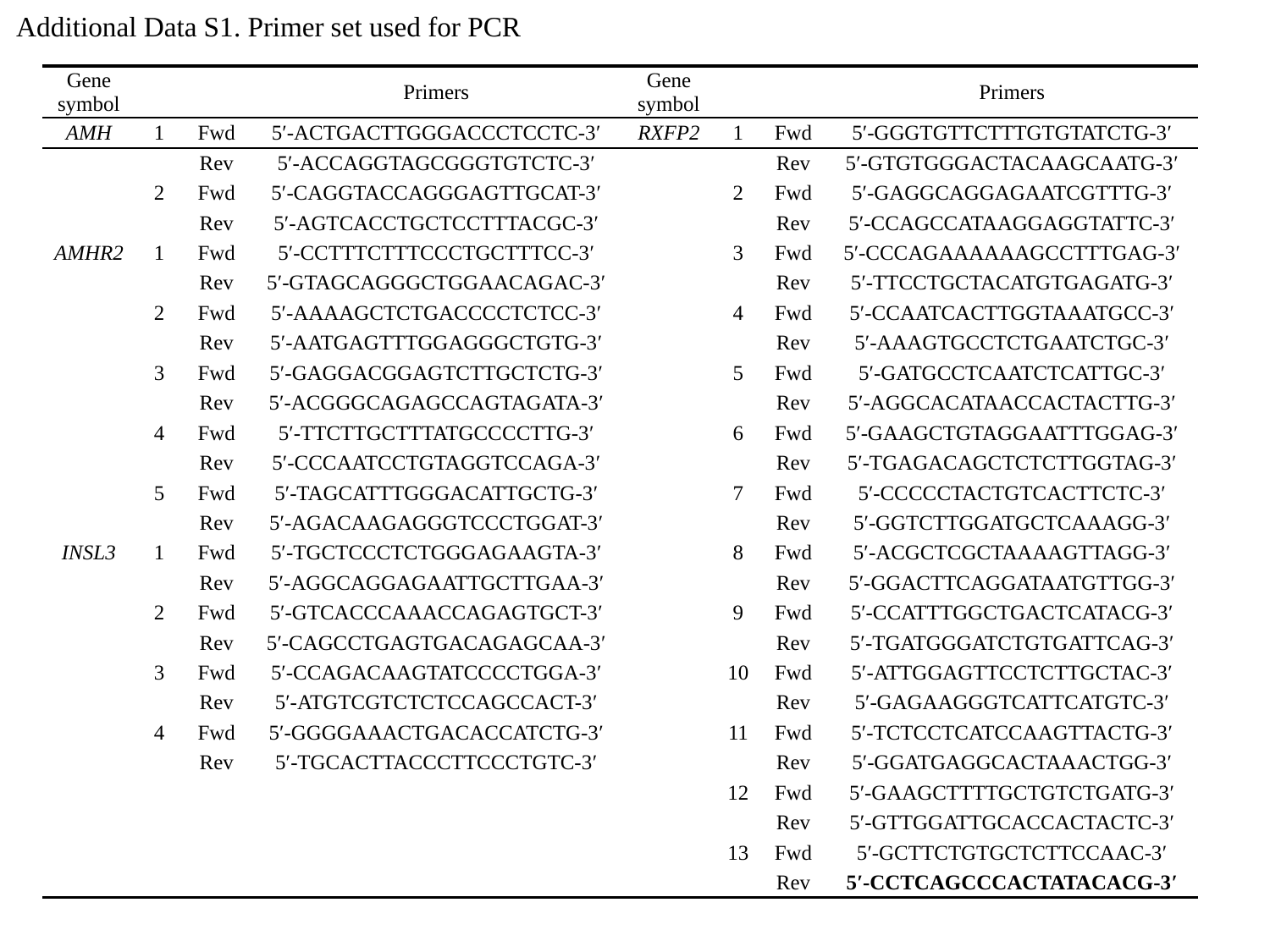

Additional Data S1. Primer set used for PCR
| Gene symbol | | | Primers | Gene symbol | | | Primers |
| --- | --- | --- | --- | --- | --- | --- | --- |
| AMH | 1 | Fwd | 5′-ACTGACTTGGGACCCTCCTC-3′ | RXFP2 | 1 | Fwd | 5′-GGGTGTTCTTTGTGTATCTG-3′ |
| | | Rev | 5′-ACCAGGTAGCGGGTGTCTC-3′ | | | Rev | 5′-GTGTGGGACTACAAGCAATG-3′ |
| | 2 | Fwd | 5′-CAGGTACCAGGGAGTTGCAT-3′ | | 2 | Fwd | 5′-GAGGCAGGAGAATCGTTTG-3′ |
| | | Rev | 5′-AGTCACCTGCTCCTTTACGC-3′ | | | Rev | 5′-CCAGCCATAAGGAGGTATTC-3′ |
| AMHR2 | 1 | Fwd | 5′-CCTTTCTTTCCCTGCTTTCC-3′ | | 3 | Fwd | 5′-CCCAGAAAAAAGCCTTTGAG-3′ |
| | | Rev | 5′-GTAGCAGGGCTGGAACAGAC-3′ | | | Rev | 5′-TTCCTGCTACATGTGAGATG-3′ |
| | 2 | Fwd | 5′-AAAAGCTCTGACCCCTCTCC-3′ | | 4 | Fwd | 5′-CCAATCACTTGGTAAATGCC-3′ |
| | | Rev | 5′-AATGAGTTTGGAGGGCTGTG-3′ | | | Rev | 5′-AAAGTGCCTCTGAATCTGC-3′ |
| | 3 | Fwd | 5′-GAGGACGGAGTCTTGCTCTG-3′ | | 5 | Fwd | 5′-GATGCCTCAATCTCATTGC-3′ |
| | | Rev | 5′-ACGGGCAGAGCCAGTAGATA-3′ | | | Rev | 5′-AGGCACATAACCACTACTTG-3′ |
| | 4 | Fwd | 5′-TTCTTGCTTTATGCCCCTTG-3′ | | 6 | Fwd | 5′-GAAGCTGTAGGAATTTGGAG-3′ |
| | | Rev | 5′-CCCAATCCTGTAGGTCCAGA-3′ | | | Rev | 5′-TGAGACAGCTCTCTTGGTAG-3′ |
| | 5 | Fwd | 5′-TAGCATTTGGGACATTGCTG-3′ | | 7 | Fwd | 5′-CCCCCTACTGTCACTTCTC-3′ |
| | | Rev | 5′-AGACAAGAGGGTCCCTGGAT-3′ | | | Rev | 5′-GGTCTTGGATGCTCAAAGG-3′ |
| INSL3 | 1 | Fwd | 5′-TGCTCCCTCTGGGAGAAGTA-3′ | | 8 | Fwd | 5′-ACGCTCGCTAAAAGTTAGG-3′ |
| | | Rev | 5′-AGGCAGGAGAATTGCTTGAA-3′ | | | Rev | 5′-GGACTTCAGGATAATGTTGG-3′ |
| | 2 | Fwd | 5′-GTCACCCAAACCAGAGTGCT-3′ | | 9 | Fwd | 5′-CCATTTGGCTGACTCATACG-3′ |
| | | Rev | 5′-CAGCCTGAGTGACAGAGCAA-3′ | | | Rev | 5′-TGATGGGATCTGTGATTCAG-3′ |
| | 3 | Fwd | 5′-CCAGACAAGTATCCCCTGGA-3′ | | 10 | Fwd | 5′-ATTGGAGTTCCTCTTGCTAC-3′ |
| | | Rev | 5′-ATGTCGTCTCTCCAGCCACT-3′ | | | Rev | 5′-GAGAAGGGTCATTCATGTC-3′ |
| | 4 | Fwd | 5′-GGGGAAACTGACACCATCTG-3′ | | 11 | Fwd | 5′-TCTCCTCATCCAAGTTACTG-3′ |
| | | Rev | 5′-TGCACTTACCCTTCCCTGTC-3′ | | | Rev | 5′-GGATGAGGCACTAAACTGG-3′ |
| | | | | | 12 | Fwd | 5′-GAAGCTTTTGCTGTCTGATG-3′ |
| | | | | | | Rev | 5′-GTTGGATTGCACCACTACTC-3′ |
| | | | | | 13 | Fwd | 5′-GCTTCTGTGCTCTTCCAAC-3′ |
| | | | | | | Rev | 5′-CCTCAGCCCACTATACACG-3′ |
