# Supplementary material for: Genetic and histopathological analysis of transverse testicular ectopia without persistent Müllerian duct syndrome: two case reports
Source: J Med Case Rep. 2020 Dec 1;14:233. doi: 10.1186/s13256-020-02559-7 (PMC7706043; doi:10.1186/s13256-020-02559-7)
Supplement: Supplementary file 2 — Additional file 2. Primer set used for sequencing [file 13256_2020_2559_MOESM2_ESM.pptx]

## Slide 1
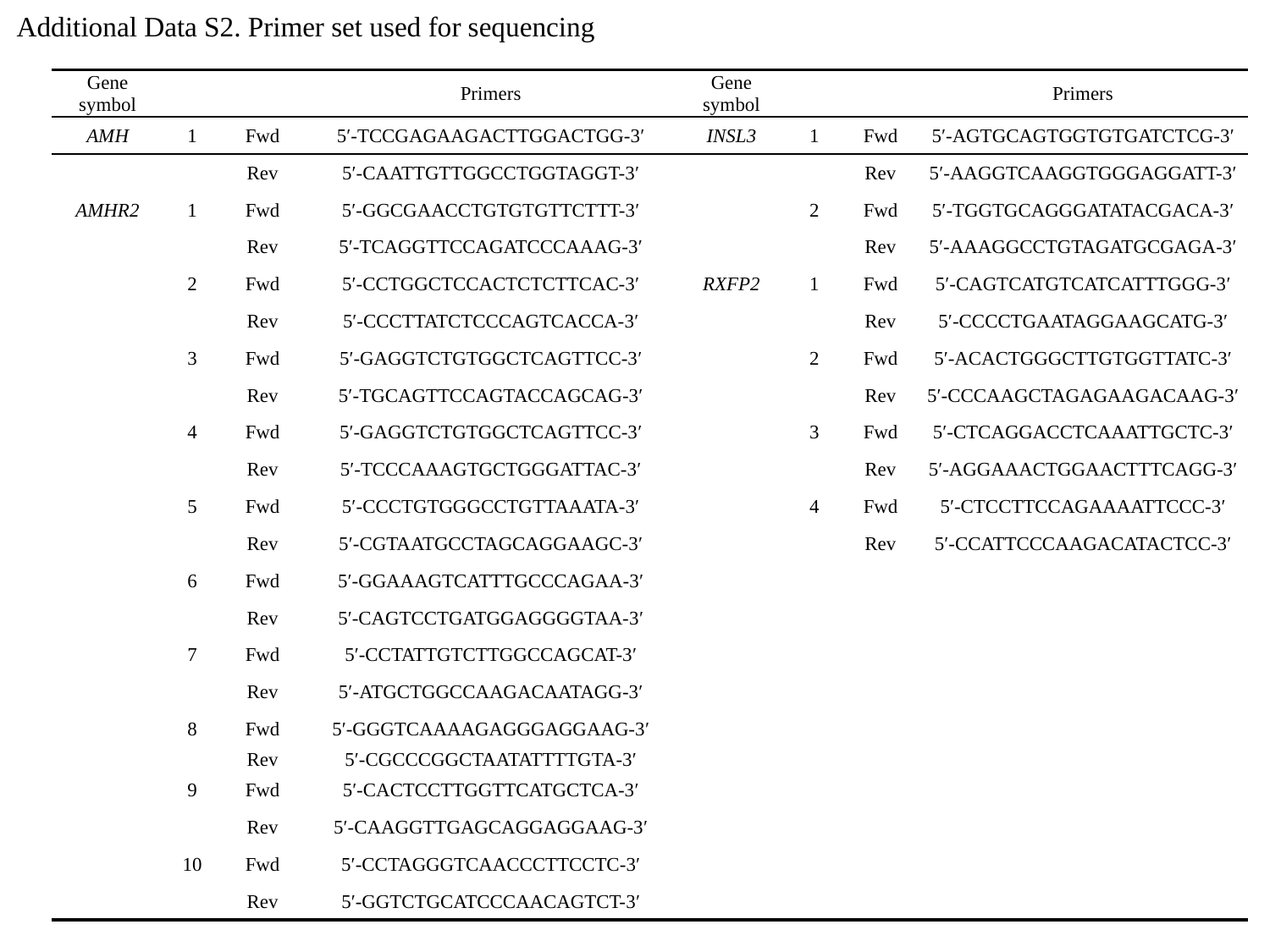

Additional Data S2. Primer set used for sequencing
| Gene symbol | | | Primers | Gene symbol | | | Primers |
| --- | --- | --- | --- | --- | --- | --- | --- |
| AMH | 1 | Fwd | 5′-TCCGAGAAGACTTGGACTGG-3′ | INSL3 | 1 | Fwd | 5′-AGTGCAGTGGTGTGATCTCG-3′ |
| | | Rev | 5′-CAATTGTTGGCCTGGTAGGT-3′ | | | Rev | 5′-AAGGTCAAGGTGGGAGGATT-3′ |
| AMHR2 | 1 | Fwd | 5′-GGCGAACCTGTGTGTTCTTT-3′ | | 2 | Fwd | 5′-TGGTGCAGGGATATACGACA-3′ |
| | | Rev | 5′-TCAGGTTCCAGATCCCAAAG-3′ | | | Rev | 5′-AAAGGCCTGTAGATGCGAGA-3′ |
| | 2 | Fwd | 5′-CCTGGCTCCACTCTCTTCAC-3′ | RXFP2 | 1 | Fwd | 5′-CAGTCATGTCATCATTTGGG-3′ |
| | | Rev | 5′-CCCTTATCTCCCAGTCACCA-3′ | | | Rev | 5′-CCCCTGAATAGGAAGCATG-3′ |
| | 3 | Fwd | 5′-GAGGTCTGTGGCTCAGTTCC-3′ | | 2 | Fwd | 5′-ACACTGGGCTTGTGGTTATC-3′ |
| | | Rev | 5′-TGCAGTTCCAGTACCAGCAG-3′ | | | Rev | 5′-CCCAAGCTAGAGAAGACAAG-3′ |
| | 4 | Fwd | 5′-GAGGTCTGTGGCTCAGTTCC-3′ | | 3 | Fwd | 5′-CTCAGGACCTCAAATTGCTC-3′ |
| | | Rev | 5′-TCCCAAAGTGCTGGGATTAC-3′ | | | Rev | 5′-AGGAAACTGGAACTTTCAGG-3′ |
| | 5 | Fwd | 5′-CCCTGTGGGCCTGTTAAATA-3′ | | 4 | Fwd | 5′-CTCCTTCCAGAAAATTCCC-3′ |
| | | Rev | 5′-CGTAATGCCTAGCAGGAAGC-3′ | | | Rev | 5′-CCATTCCCAAGACATACTCC-3′ |
| | 6 | Fwd | 5′-GGAAAGTCATTTGCCCAGAA-3′ | | | | |
| | | Rev | 5′-CAGTCCTGATGGAGGGGTAA-3′ | | | | |
| | 7 | Fwd | 5′-CCTATTGTCTTGGCCAGCAT-3′ | | | | |
| | | Rev | 5′-ATGCTGGCCAAGACAATAGG-3′ | | | | |
| | 8 | Fwd | 5′-GGGTCAAAAGAGGGAGGAAG-3′ | | | | |
| | | Rev | 5′-CGCCCGGCTAATATTTTGTA-3′ | | | | |
| | 9 | Fwd | 5′-CACTCCTTGGTTCATGCTCA-3′ | | | | |
| | | Rev | 5′-CAAGGTTGAGCAGGAGGAAG-3′ | | | | |
| | 10 | Fwd | 5′-CCTAGGGTCAACCCTTCCTC-3′ | | | | |
| | | Rev | 5′-GGTCTGCATCCCAACAGTCT-3′ | | | | |
